# Decosus: An R Framework for Universal Integration of Cell Proportion Estimation Methods

**DOI:** 10.3389/fgene.2022.802838

**Published:** 2022-04-01

**Authors:** Chinedu A. Anene, Emma Taggart, Catherine A. Harwood, Daniel J. Pennington, Jun Wang

**Affiliations:** ^1^ Centre for Cancer Genomics and Computational Biology, Barts Cancer Institute, Queen Mary University of London, London, United Kingdom; ^2^ Centre for Cancer Biology and Therapy, School of Applied Science, London South Bank University, London, United Kingdom; ^3^ Centre for Immunobiology, Barts and the London School of Medicine, Blizard Institute, Queen Mary University of London, London, United Kingdom; ^4^ Centre for Cell Biology and Cutaneous Research, Barts and The London School of Medicine and Dentistry, Blizard Institute, Queen Mary University of London, London, United Kingdom; ^5^ Department of Dermatology, The Royal London Hospital, Barts Health NHS Trust, London, United Kingdom

**Keywords:** cell deconvolution, R package, method integration, gene expression, immuno-biology

## Abstract

The assessment of the cellular heterogeneity and abundance in bulk tissue samples is essential for characterising cellular and organismal states. Computational approaches to estimate cellular abundance from bulk RNA-Seq datasets have variable performances, often requiring benchmarking matrices to select the best performing methods for individual studies. However, such benchmarking investigations are difficult to perform and assess in typical applications because of the absence of gold standard/ground-truth cellular measurements. Here we describe Decosus, an R package that integrates seven methods and signatures for deconvoluting cell types from gene expression profiles (GEP). Benchmark analysis on a range of datasets with ground-truth measurements revealed that our integrated estimates consistently exhibited stable performances across datasets than individual methods and signatures. We further applied Decosus to characterise the immune compartment of skin samples in different settings, confirming the well-established Th1 and Th2 polarisation in psoriasis and atopic dermatitis, respectively. Secondly, we revealed immune system-related UV-induced changes in sun-exposed skin. Furthermore, a significant motivation in the design of Decosus is flexibility and the ability for the user to include new gene signatures, algorithms, and integration methods at run time.

## Introduction

Gene expression quantification is indispensable for the interrogation of cellular and organismal states. However, bulk tissue samples of interest in clinical research have considerable cellular heterogeneity that standard methods (Microarray and RNA-Seq) cannot decipher. Although single-cell technologies have been developed to uncover the cellular heterogeneity within cell populations, they have a range of limitations (e.g., time, tissue types, dropouts, technical noise, and cost), making large-scale or clinical applications impossible. Since bulk analysis methods report average expression levels, it is often challenging to disentangle changes in cell-type composition from fundamental differences in states. To address this, several computational tools (so-called deconvolution methods) have been developed to estimate cell-type composition within bulk expression data. These utilise various models, including least squares regression (Abbas et al., 2009), constrained least squares regression (Li et al., 2016; Racle et al., 2017; Finotello and Trajanoski, 2018), quadratic programming (Gong et al., 2011; Gong and Szustakowski, 2013; Zhong et al., 2013), support vector regression (Newman et al., 2015), the geometric mean of marker gene expression (Becht et al., 2016) and single-sample gene set enrichment analysis (ssGSEA) (Aran et al., 2017), extensively reviewed in (Sturm et al., 2019).

Unfortunately, individual tools have limitations that affect their effective utilisation. Critically, due to the differences in the underlying statistical assumptions and marker gene signatures, the approaches often produce different results in a data type-dependent manner (Jiménez-Sánchez et al., 2019; Sturm et al., 2019). Thus, there is a need for a consensus approach that can combine and integrate these methods into a single robust output.

Recent benchmarking studies (Jiménez-Sánchez et al., 2019; Sturm et al., 2019) have provided robust frameworks to systematically integrate the outputs of these methods into a single unified deconvolution solution, thereby reducing the limitations of individual methods. While these approaches are helpful, significant challenges remain. Jiménez-Sánchez et al., restricted their implementation to a set of common cancers (Jiménez-Sánchez et al., 2019), while Sturm et al., focused on method performance on key immune cell types (Sturm et al., 2019). Thus, neither tool can be applied universally, especially for poorly studied cancers, and non-cancer conditions or deconvolution of non-immune cells. We developed a deconvolution integration tool (Decosus, available as an R package) without tissue type restrictions, allowing for universal application of the integration framework. A further advantage of Decosus is the ability to generate both relative (allowing sample-to-sample comparison only) and absolute (also allowing within-sample comparison of one cell type to another) estimations where possible. We demonstrate the utility of Decosus through reference datasets and use cases, including cancer and non-cancer datasets. Decosus generates a validated and robust decomposition of cell composition in various tissue types while allowing for flexible integration of new methods and signatures as they become available (github.com/caanene1/Decosus).

## Materials and Methods

### Deconvolution Method

We used common cell deconvolution methods and signatures to estimate cell composition from bulk RNA-Seq datasets individually. The methods include EPIC (Racle et al., 2017), MCPcounter (Becht et al., 2016), quanTISeq (Finotello et al., 2019), and xCell (Aran et al., 2017). The estimates for the four methods were generated using the corresponding R implementations: MCPcounter (v1.2.0), EPIC (v1.1.5), xCell (v1.1.0), and quanTISeq (github.com/icbi-lab/quanTIseq). In addition to these, we used the following gene signatures: Danaher, Davoli, Rooney (Bindea et al., 2013; Danaher et al., 2017; Davoli et al., 2017), and averaged the gene expressions to provide measures of cellular abundance for each signature. Additionally, we provide optional gene signatures from the CellMarker database (Zhang et al., 2019) and use this in the analyses featured here.

### Decosus Framework

Decosus is an R package that flexibly integrates the estimates of cell compositions from deconvolution methods and cell signatures. We identified cell types for which estimates exist in at least two of the seven default methods and signatures (Supplementary S1 CSV). This mapping was similar to that of a previously published method, Consensus^TME^, where cells were exhaustively mapped to a controlled vocabulary of cell types (Jiménez-Sánchez et al., 2019). However, in Decosus, the user can expand the consensus through the optional arguments for new signatures and mappings (https://github.com/caanene1/Decosus), ensuring that Decosus can produce results relevant to the most up-to-date cell signatures and allowing the addition of rarer cell types. For instance, licensing restrictions did not allow us to include CIBERSORT into the package, but a user can still integrate CIBERSORT if you obtain the output or source code from the author’s website.

After generating the estimates for the individual methods, we average the values for the source cells to create a single estimate for the cell types in the controlled vocabulary (Supplementary S1 CSV). The assumption behind this approach is that it limits any one method from dominating the estimates, thereby ensuring the consensus is closer to the best measure or is the best performing measure (see results section). We also included optional arguments in the function call to specify whether the data is to be scaled or not and the aggregation mode (i.e., mean (default) or geometric mean). Furthermore, two outputs are provided; 1) relative, the default using all methods, and 2) absolute, which is derived by limiting the methods used to those that can be considered absolute cell compositions (EPIC and quanTISeq, reviewed previously by Sturm et al., 2019). This option offers a less comprehensive selection of consensus cells than the relative output but may be helpful in analyses requiring cell-cell comparison, which is not permitted when using all seven algorithms due to the methods involved (see Table 1 and Sturm et al., 2019). The Decosus R package can be obtained through GitHub (https://github.com/caanene1/Decosus).

**TABLE 1:** Default deconvolution method and signatures included in Decosus.

Signature/Method	Type	Comparison
Xcell	Algorithm	Across Samples
MCP-counter	Algorithm	Across Samples
quanTISeq	Algorithm	Across Samples & Between Cells
EPIC	Algorithm	Across Samples & Between Cells
Danaher	Signature	Across Samples
Davoli	Signature	Across Samples
Rooney	Signature	Across Samples

### Datasets for Benchmarking

To benchmark the Decosus framework, we obtained pre-processed bulk RNA-seq and FACS data from Sturm et al. (2019) through their Github repository (https://github.com/icbi-lab/immune_deconvolution_benchmark/releases/download/v1.0.0-rcl/data.tar.gz). This data includes eight healthy PBMC samples (Hoek data) (Finotello et al., 2019), four metastatic melanoma samples (Racle data) (Racle et al., 2017) and three ovarian cancer ascites samples (Schelker data, x2 replicates) (Schelker et al., 2017). We obtained 20 PBMC samples analysed by microarray (gene-level values) and flow-cytometry from the CIBERSORT web portal (Cibersort data) (Newman et al., 2015). Additionally, we obtained the two (SDY311, *n* = 76; SDY420 ref, and *n* = 105) pre-processed bulk RNA-seq and FACS data from the Immport study (Alpert et al., 2019) through the xCell Github repository (https://github.com/dviraran/xCell/tree/master/vignettes).

To evaluate the performance of Decosus in real-world clinical contexts, we interrogated two additional RNA-Seq datasets. The first dataset contained skin samples of atopic dermatitis (*n* = 54) and psoriasis (*n* = 55) (GSE121212) (Tsoi et al., 2019). The second dataset was normal skin samples (sun-exposed and non-sun exposed) samples from the GTEX project (*n* = 1879) (Lonsdale et al., 2013).

All RNA-Seq gene expression values are expressed as transcript per million (TPM).

### Datasets for Simulation Studies

To evaluate how well Decosus estimates known cell compositions compared to individual methods, we simulated cell mixtures from single-cell RNA-Seq datasets. Specifically, we extracted the RNA expression values for select samples from the Panglaodb data portal (Franzén et al., 2019), including SRA701877, SRS3279685, SRA713577, SRS3363004, SRA716608, SRS3391633, SRA779509, SRS3805246, SRA878024, and SRS4660846.

Next, we generated expression profiles as below:

1 Given a dataset of annotated n single cell types, assign random fractions to each cell type (the fractions sum to 100 and integers) (data 1).
2 Generate an expression matrix of single cells with 100 columns by randomly selecting the corresponding fraction of the available samples for the selected cell types to be included in the matrix. Here, we used a random selection to introduce noise like the variation in real datasets (data 2). We allow sampling with replacement if the cell type-specific fraction is bigger than the available single cells.
3 Finally, generate a simulated expression profile by adding the expression values across the rows of data 2 and use data 1 as the ground truth.

We repeat the process 500 times (per data source) with different fractions, samples, and cell types.

## Results

### Overview of the Decosus Integration Framework

Our framework integrates seven deconvolution algorithms and cell signatures into consensus estimates of cell composition (Table 1). We do this by selecting and averaging the shared cell types across the tools (Supplementary S1 CSV). It requires a gene expression dataset and a set of optional parameters under a single R function (set to reasonable defaults, see github. com/caanene1/Decosus). The R implementation of the framework allows for flexible inclusion of new algorithms or signatures at run time (see https://github.com/caanene1/Decosus). When available, the final output has two tables representing relative and absolute consensus estimates (see *Materials and Methods*).

### Analysis of Simulated Cell Proportions Demonstrate the Stability of Decosus

**FIGURE 1:**
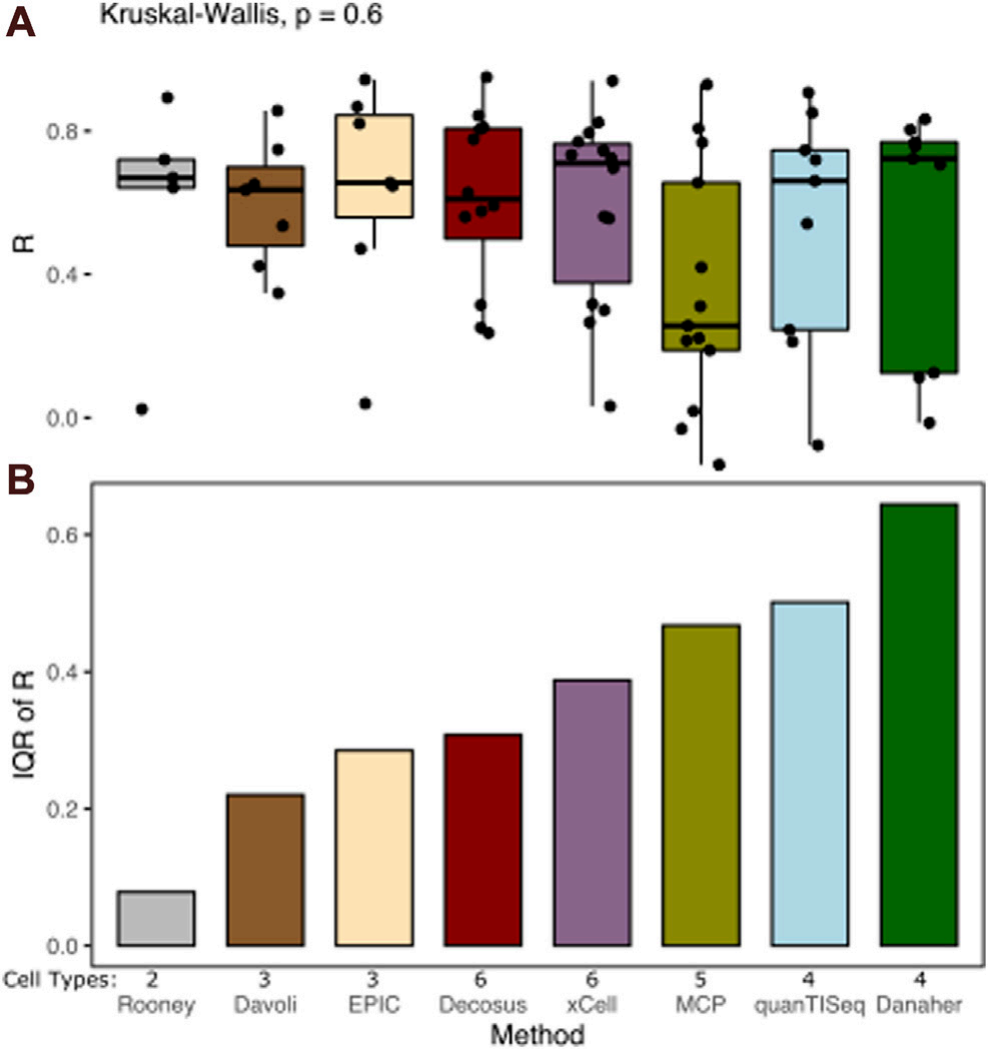
Decosus produces stable estimates of cell proportions. **(A)** Box plot of the Pearson’s R of correlation between decomposed and simulated cell fractions (*n* = 500 per single-cell RNA-Seq data used in the simulations studies). The higher the R, the more similar the decomposed and simulated cell fractions are. **(B)** Bar plot of inter-method variability in Pearson’s R of correlation between decomposed and simulated cell fractions measured by IQR. The lower the IQR, the more stable the estimates from the method across datasets and cell types. Within the plots, colours represent the method of estimating cell fractions from bulk RNA-Seq datasets. The number of cell types covered for each method in the simulation is indicated (Cell Types). MCP = MCPcounter.

Cell proportion deconvolution methods produce different results in a data type-dependent manner. Decosus combines and integrates these methods into a single stable consensus value to reduce the data-dependent differences in performance. To assess how well the framework achieves this aim, we first evaluated its ability to decompose known cell proportions using simulated gene expression profiles (see Methods). We simulated large sets of bulk-expression profiles (n = 2,500) with specific cell proportions from multiple single-cell RNA-Seq datasets (source, n = 5). We used multiple sources to reflect data-specific differences in real applications. Interrogating the correlation coefficient between the estimates and the expected cell proportions across a range of cell types revealed surprisingly stable estimates of cell proportions (Figure 1). Decosus had the 4th lowest interquartile range (IQR = 0.31) compared to xCell (IQR = 0.39), MCPcounter (IQR = 0.47), quanTISeq (IQR = 0.50), and Danaher (IQR = 0.64) (Figure 1A). Although Rooney (IQR = 0.09), Davoli (IQR = 0.22), and EPIC (IQR = 0.29) were lower than Decosus, this is potentially due to the small number of cell types they covered in the simulation (Davoli = 3, EPIC = 3, and Rooney = 2), compared to the other methods (Decosus = 6, xCell = 6, MCPcounter = 5, quanTISeq = 4, and Danaher = 4). Indeed, normalising for the number of cell types revealed Decosus has the second-lowest IQR (Supplementary Figure S1). The stability of Decosus estimates is due to the robustness of averaging multiple signatures and methods (see Methods). As expected, there is no difference between median R values across the methods (Kruskal–Wallis, *p* = 0.6, Figure 1B), suggesting that Decosus increases the stability of the estimates without reducing the average performance expected from individual tools.

### Benchmark on Known Cell Proportions Demonstrate the Utility of Decosus

**FIGURE 2:**
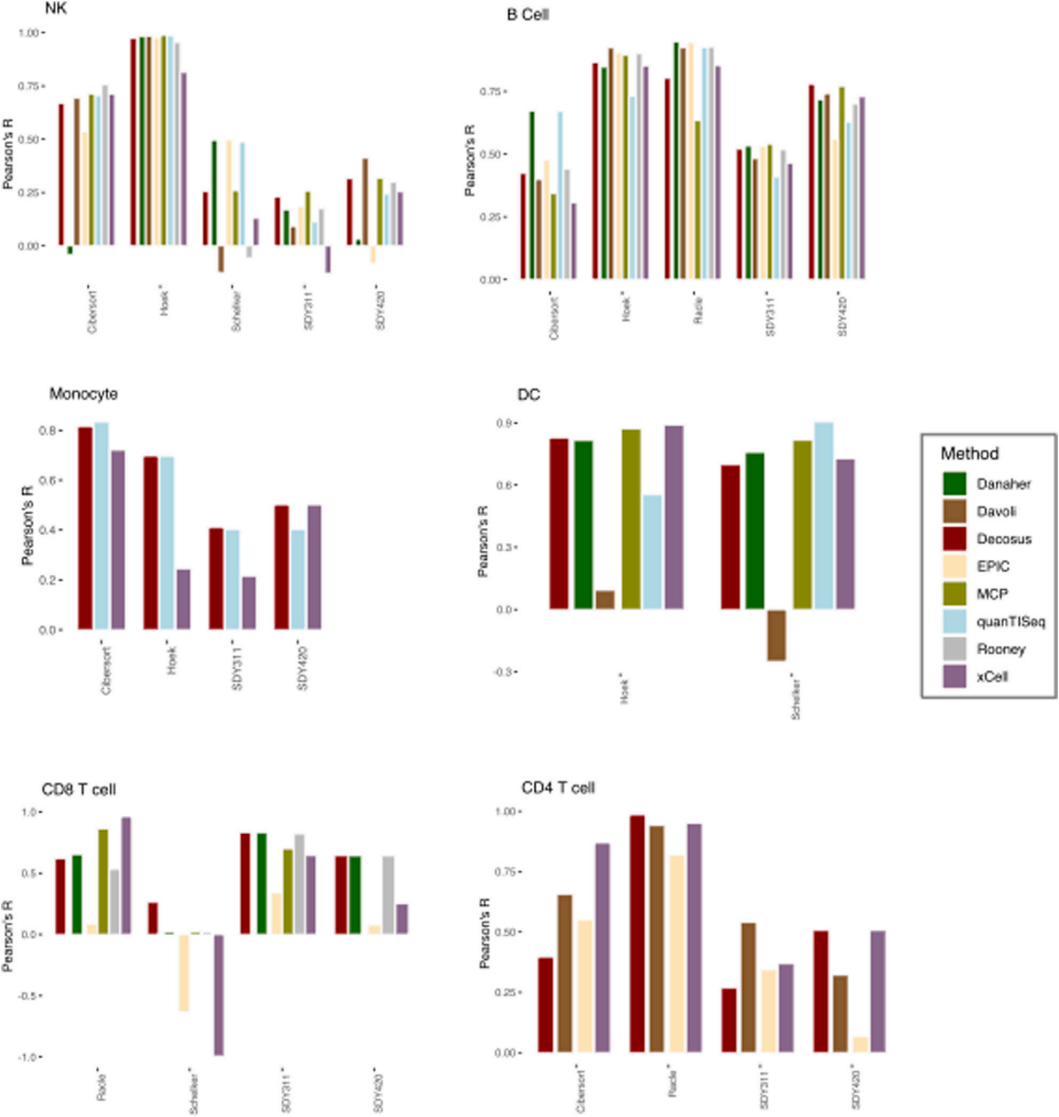
Decosus identifies known cell fractions. Bar plots of the Pearson’s R of the correlation between computer-generated cell fractions and flow cytometry fractions. The higher the R, the more similar is the computational estimates and the gold standard. Within the plots colours represent the method of estimating cell fractions from bulk RNA-Seq datasets. MCP = MCPcounter.

We applied the Decosus framework to six benchmark datasets (see Methods) and compared the estimated proportions with their corresponding flow cytometry fractions used as ground-truth. We also interrogated the distribution of the performance statistic across the individual methods and datasets. To ensure we can perform meaningful comparative analysis, we restricted our analysis to six cell types (natural killer cells, dendritic cells, monocytes, CD4^+^ T-cells, CD8^+^ T-cells and B-cells) present in two or more ground truth datasets. Our consensus estimates obtained a high correlation with the flow cytometry fractions (Median R = 0.64) across all datasets and the evaluated cell types (Figure 2). This observation was consistent with the contributing methods/signatures being highly concordant with the corresponding gold standards (Figure 2). However, we observed unpredictable performance differences across the same cell type for the individual methods (Figure 2). For natural killer (NK) cells, Danaher (r = −0.04038) and EPIC (r = 0.53) had the worst performances in the Cibersort dataset, Rooney (r = −0.0557), xCell (r = 0.129) and Davoli (r = −0.125) in Schelker, and EPIC (r = −0.08), Danaher (r = 0.0316), quanTISeq (r = 0.243), and xCell (r = 0.253) in SDY420, while all the methods performed well in Hoek or poorly in SDY311 (Figure 2). We observed similar behaviour for monocytes, where xCell performed worse in Hoek (r = 0.244) and xCell (r = 0.215) in SDY311. Interestingly, for dendritic cells, Davoli performed worse in both Hoek (r = 0.092) and Schelker (r = 0.25), but QuanTISeq additionally performed poorly in Hoek datasets (r = 0.55). Although T and B cells have multiple subtypes making benchmark analysis difficult, we generally made similar observations for CD4^+^ T cells, CD8^+^ T cells and B cells (Figure 2), where the different combinations of methods performed worse in different datasets. These observations suggest that no single method can guarantee top performance across user cases, even for the same cell type. However, our consensus estimates consistently performed well across the datasets, regardless of cell types and user cases (Figure 2). For instance, it had high performance in all datasets with DC (Hoek, r = 0.82; Schelker, r = 0.69) and most datasets with NK cells (Cibersort, r = 0.668; Hoek, r = 0.973; SDY420, r = 0.315). Furthermore, Decosus was able to derive the cellular estimates for all six cell types benchmarked here, whilst other methods/frameworks were restricted to a subset of cell types where marker gene signatures were included internally (Figure 2).

### Decosus Identified the Immunological Differences Between Atopic Dermatitis and Psoriasis

**FIGURE 3:**
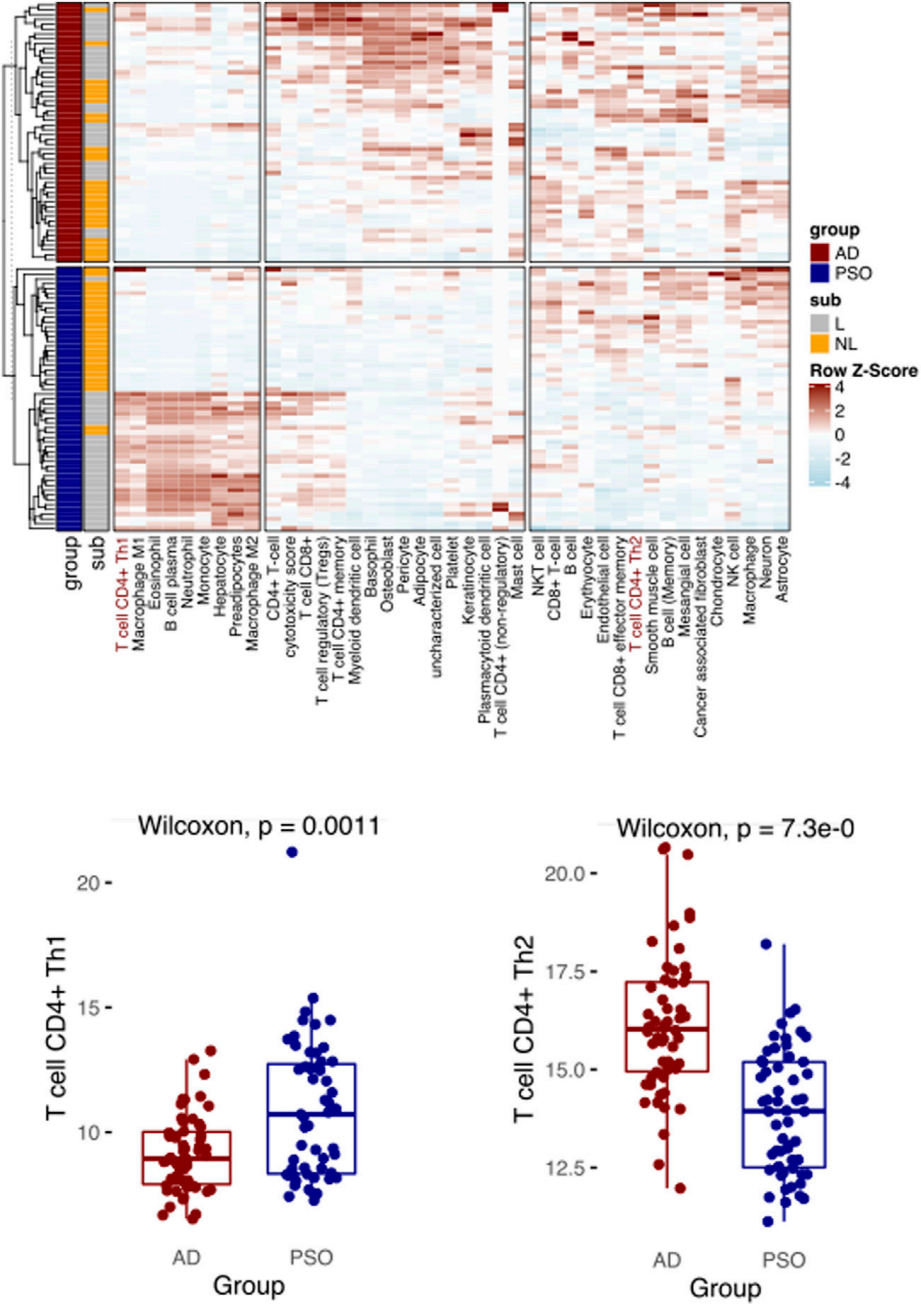
Decosus identifies the Th1 and Th2 differences between atopic dermatitis and psoriasis. (Top) Heatmap of Decosus estimated cell fractions across atopic dermatitis (AD, *n* = 54) and psoriasis (PSO, *n* = 55) samples from GSE121212. Within the plot, Th1 and Th2 cells are indicated in red, while other cells of interest are highlighted with blue. (Bottom) Box plots of the Decosus estimates of Th1 cells (left) and Th2 (right) fractions across skin samples of AD and PSO. Multiple test adjusted T. test *p* values are indicated. Within the heatmap, NL = non-lesional and L = lesional.

To further demonstrate the utility of Decosus and evaluate its performance in other physiological and health settings, we focused on diseases and cell types that were not well covered and annotated by existing methods. Here, we simulated a condition with known condition-dependent differences in cell proportions. Specifically, atopic dermatitis (AD) and psoriasis (PSO) are common skin conditions associated with barrier dysfunction. Both are characterised by T-cell driven inflammation; however, in AD, CD4^+^ T helper cells (Th) are polarised towards a Th2 phenotype, while Th1 polarization is characteristic of PSO (Brunner et al., 2017; Albanesi, 2019). Thus, immune cell composition estimated from bulk RNA expression profiles from PSO and AD skin samples should enrich for Th1 and Th2 cell signatures, respectively. Note that many existing methods do not have these two cell types, thus could not handle such cases. To this end, we collected 54 AD and 55 PSO samples from a publicly available dataset (GSE121212) (Tsoi et al., 2019) and applied our framework to interrogate the enriched immune profiles. We observed a significantly higher Th1 cell signature in PSO samples than AD samples and the reverse for Th2 (Figure 3), aligning with what is widely recognised in the literature (Brunner et al., 2017; Albanesi, 2019). The results also showed expansions in other cell types known to infiltrate each lesion, such as basophils in AD (Mashiko et al., 2017), macrophages and neutrophils in PSO (Lowes et al., 2014) (Figure 3). These results indicate that our framework can provide robust estimates of cell proportions in non-cancer bulk tissue samples, like precursor lesions.

### Decosus Enables the Identification of UV Mediated Immune Reprogramming in the Skin

**FIGURE 4:**
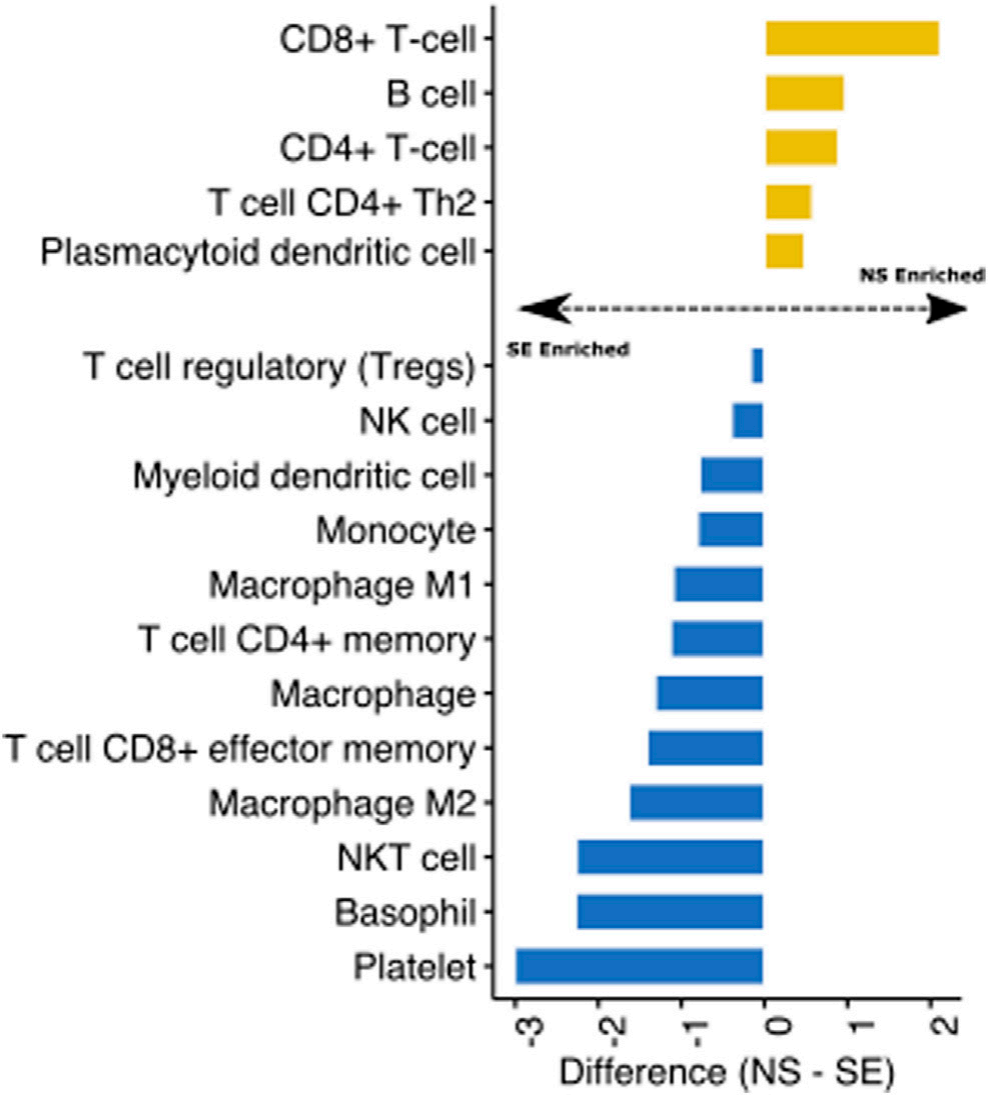
Identification of UV-mediated reprogramming of skin immune cell profile. Bar plot depicting the mean difference between the Decosus immune cell estimates for non-sun exposed skin (NS) and sun-exposed skin (SE) (n = 1879). The plot shows only immune cells significantly different between the two groups at *p*-value < 0.05.

Finally, we utilised transcriptomic data from the GTEX project of non-sun exposed and sun-exposed skin to evaluate the impact of ultraviolet radiation (UV) on skin immune profiles. We assessed the difference between the immune cell estimates of the sample groups and visualised the fold change for significant (T-test, *p* < 0.05) cell types. We found that UV exposure significantly enriched several immune cell types in the skin, including monocytes, dendritic cells, and macrophages (Figure 4). Interestingly, CD4^+^ and CD8^+^ T cells were depleted in sun-exposed skin (Figure 4). These observations are consistent with previous studies showing that UV exposure inhibits the expansion of these T cell subtypes while increasing innate immune cells (Rana et al., 2008; Gläser et al., 2009). Reprogramming of T cell composition is consistent with the idea that impaired immune function through UV damage plays a role in skin cancers (Freeman et al., 2014; Slater and Googe, 2016). Indeed, active research programmes in our group are using Decosus to help characterise the immunological factors underlying the progression of actinic keratoses (sun-damaged skin, pre-malignant lesions) to squamous cell carcinomas.

## Discussion

Estimating the cellular compositions in bulk RNA-Seq samples has been addressed by various algorithms and gene signatures. These approaches have different assumptions and strengths, frequently resulting in varied performances across the different dataset (Sturm et al., 2019). Thus, it is challenging to objectively select the best method in real applications which lack ground truth cell fractions. The Decosus framework addressed this gap by providing consensus estimates that exhibit consistent performance across different benchmark datasets and has further invaluable features not found in existing consensus methods. The main utilities of Decosus are that it 1) leverages the strengths of the individual methods and signatures while minimising their weakness, and 2) provides a broader coverage of cell types.

Further,our framework is inherently expandable, whereby the user can add new methods, signatures, and cell mappings to the default set. We demonstrated this function by including gene sets from the CellMarker database to enable the consensus estimation of Th1 and Th2 cell fractions in AD and PSO samples (Figure 2). This flexible approach is critical for robustly estimating cell abundance and fractions across biological states and represents an important advance in the domain compared to previous methods that exclusively focus on common cancers or cell types. Though the expansion of the signatures and mappings may inadvertently incorporate spurious estimates to the consensus, the multiple sources per cell type can better capture the diversity in cellular gene expression profiles across different biological states. Unlike the Consensus^TME^ that generated new consensus gene signatures (Jiménez-Sánchez et al., 2019), we solved this problem by first calculating the cell estimations individually for each method (see *Methods*) before taking the average value for the same cell in each method. Here, a future update may include additional ways to generate consensus estimates such as geometric mean, trimmed and weighted averages, particularly as validation datasets become available. Further advantages to our tool are the ability to apply Decosus to any tissue type or disease compared to previous efforts to create consensus cell composition estimates, which focused on tumour tissues (Jiménez-Sánchez et al., 2019; Sturm et al., 2019). We also incorporated the option to use absolute estimates, which is crucial for applications requiring cell-cell comparisons.

Benchmarking on the PBMC and cancer datasets showed that although each method ranked highly in at least one dataset, we saw highly varied correlations across datasets. This performance issue is expected but impossible to identify in actual use cases because of the differences in the statistical assumptions and gene signatures associated with each method. Our consensus estimates reduce this unpredictable behaviour by averaging out the poorly performing methods. Indeed, we adequately identified the expected Th1 cells enriched in PSO and the Th2 cells in AD. It is worth noting that many existing methods, such as Cibersort, EPIC, and ConsensusTME, could not resolve such a case due to their limited coverage of diseases/conditions or cell types (see Supplementary S2 CSV), further highlighting the versatility of our method.

An important limitation of the Decosus framework is that it represents the aggregate performance of the contributing methods. Thus, if they have universally poor performance for a given case, then Decosus will have a corresponding poor performance. For instance, all the approaches, including Decosus, performed poorly in decomposing NK cells and Monocytes from the SDY311 dataset (Figure 2). However, Decosus is stable for most cases compared to the individual methods.

To allow for easy incorporation of Decosus into new and existing workflows, we implemented an object-oriented system in R, allowing the user to add, retrieve and evaluate individual methods (https://github.com/caanene1/Decosus). The full output of Decosus provides consensus estimates when available and offers unified interphase for the procedures. Although, Decosus is implemented and valid for human data, the framework can easily be expanded with new methods and signatures, including for other species at run time, as demonstrated in the analysis of Th1 and Th2 cells in skin samples. Moreover, one of the future directions of Decosus is to create a flexible function within our method that allows users to input associated weights of individual methods and additive equations, to facilitate users to infer the most accurate estimates of cellular compositions in their biological settings. However, this will require very large validation datasets to derive weights accurately for the biological setting of interest.

## Data Availability

The original contributions presented in the study are included in the article/Supplementary Material, further inquiries can be directed to the corresponding author.

## Supplementary Material

Supplementary Figure 1 Bar plot of inter-method variability in Pearson’s R of correlation between decomposed and simulated cell fractions measured by IQR normalised by the number of cell types covered for each method in the simulation. MCP = MCPcounter.
