# Supplementary figures and images for: Decosus: An R Framework for Universal Integration of Cell Proportion Estimation Methods
Source: Front Genet. 2022 Apr 1;13:802838. doi: 10.3389/fgene.2022.802838 (PMC9011041; doi:10.3389/fgene.2022.802838)

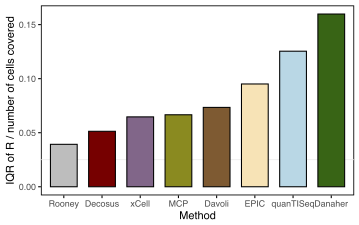

Supplement: Supplementary file 1 [file Image1.tiff]
